# SpSrtA-Catalyzed Isopeptide Ligation on Lysine Residues

**DOI:** 10.3390/microorganisms12010179

**Published:** 2024-01-17

**Authors:** Jiajia Wu, Tianyu Chu, Jian Hao, Liang Lin

**Affiliations:** 1 Department of Chemistry, Shanghai University, Shanghai 200444, China; 2 State Key Laboratory of Chemical Biology, Shanghai Institute of Organic Chemistry, Chinese Academy of Sciences, Shanghai 200032, China; 3 College of Chemistry and Molecular Engineering, Peking University, Beijing 100871, China

**Keywords:** isopeptide ligation, sortase A, yeast display

## Abstract

Sortase-mediated ligation (SML) is widely used for protein bioconjugation. However, the sortase used in this strategy typically recognizes only the N-terminal oligoglycine, which is absent in most natural proteins. To broaden the spectrum of substrates compatible with SML, we focus on a novel sortase, sortase A from *Streptococcus pneumoniae* (SpSrtA), known for its expanded substrate specificity (N-terminal glycine, alanine, and serine). We present the first evidence showing that the reported SpSrtA mutant (SpSrtA*) can modify lysine residues in itself and other proteins. The modification sites of SpSrtA* were identified through LC-MS/MS analysis. Moreover, we discovered an optimal lysine-containing peptide tag by fusing it onto sfGFP, resulting in a labeling efficiency of 57%. Inspired by this, we applied the method to modify proteins on microorganism surfaces up to 13.5-fold. To enhance labeling efficiency, we fused the SpSrtA* onto a surface protein and achieved a 2.64-fold improvement. We further developed a high-throughput yeast display screening method for the directed evolution of SpSrtA*, achieving a 10-fold improvement in the labeling efficiency of this surface protein. Our study provides a novel strategy for modifying the lysine residues that will be a powerful addition to the protein bioconjugation toolbox.

## 1. Introduction

Protein–protein ligation, a crucial technique in biochemistry and molecular biology, involves the covalent attachment of specific side chains of amino acid residues in proteins, such as −NH2 in lysine residue, −SH in cysteine residue, −OH in serine/threonine/tyrosine residue, and N-/C-terminus [1,2,3]. However, traditional methods lack chemoselectivity, leading to the formation of heterogeneous conjugates, which can adversely affect protein functionality and stability [4]. Sortase-mediated ligation (SML), in which two moieties are efficiently conjugated at their termini, has emerged as a potent and chemoselective strategy [5]. Sortase A (SrtA), a transpeptidase from Gram-positive bacteria, exhibits specificity for the LPXTG motif (X represents any amino acid). It cleaves the amide bond between threonine and glycine through a nucleophilic cysteine, generating a stable acyl–enzyme intermediate [6]. In canonical mechanisms, this intermediate undergoes nucleophilic attack by peptides or proteins with N-terminal oligoglycine, leading to transpeptidation [7,8]. However, the N-terminal oligoglycine is uncommon in natural proteins, requiring additional complex modifications for SML [9,10]. Consequently, there is a necessity to expand the range of SrtA nucleophilic substrates compatible with SML for protein bioconjugation.

Given its nucleophilicity, solvent accessibility, and relative abundance, lysine residues act as prime targets for chemoselective protein bioconjugation [11,12]. Various protein ligases, such as SpyLigase [13], SnoopLigase [14], transglutaminase [15], and biotin ligase [16], have been developed to target lysine residues. Despite the efficiency of these methods, current methods typically recognize only a limited range of lysine-containing sequences. While SrtA can catalyze the formation of isopeptide bonds by modifying lysine side chains in natural pilin motifs, its application to universal lysine residues remains unexplored [17,18]. Recently, a triple-mutant *Streptococcus pneumoniae* SrtA (E189H/V206I/E215A, referred to as SpSrtA* in this study) [2] has shown potential in modifying a broader array of lysine residues. Distinct from common SrtA, SpSrtA* is calcium-independent and accepts multiple N-terminal residues as nucleophiles, including glycine, alanine, and serine [2]. Notably, SpSrtA* exhibits a larger substrate recognition site conducive to accommodating lysine residues [19,20,21,22]. 

In this study, we introduce SpSrtA* as a novel tool for the universal modification of lysine residues. We initially established that SpSrtA* could utilize its own lysine residues as nucleophilic substrates. Most proteins containing lysine residues can also be modified. Moreover, we screened out an optimal lysine-containing peptide tag derived from SpSrtA*, which was found to significantly enhance labeling efficiency when fused with this tag. This tool was further applied to modify proteins on microorganism surfaces. While there was limited success in enhancing labeling efficiency by fusing SpSrtA* onto surface proteins, we ultimately engineered variants of SpSrtA* to significantly improve labeling efficiency for the target surface proteins. 

## 2. Materials and Methods

### 2.1. Reagents and Materials

The peptide biotin-LPETGRRR-*NH_2_* (98.4%) was purchased from BankPeptide (Anhui, China) and *Abz*-LPETGK(Dnp)-*NH_2_* (95%) was purchased from AnaSpec (Fremont, CA, USA). The antibodies used in this study were Streptavidin Alexa Fluor™ 488 (S11223, Invitrogen, Waltham, MA, USA), Goat anti-mouse IgG (H+L) highly cross-adsorbed secondary antibody, Alexa Fluor™ 647 (A-21236, Invitrogen, Waltham, MA, USA), streptavidin-HRP (3999S, Cell Signaling Technology, Danvers, MA, USA), anti-GFP antibody (sc-9996, Santa Cruz Biotechnology, Dallas, TX, USA), anti-V5 antibody (B1005, Biodragon, Suzhou, China), and HRP-conjugated goat anti-mouse secondary antibody (BF03001, Biodragon, Suzhou, China). 

### 2.2. Recombinant Protein Expression and Purification

Genes of various recombinant proteins, including sfGFP (Addgene ID: 102634), MBP (NCBI ID: 7MN5_B), SpyCatcher (NCBI ID: 4MLI_A), GST (NCBI ID: 1BG5_A), and SpSrtA* (NCBI ID: 3FN5_A, E189H/V206I/E215A [2]), were cloned into pET-28a (+) vector using *NcoI* and *XhoI* restriction sites. These recombinant plasmids were subsequently expressed in *E. coli* BL21 (DE3) cells. Protein expression was induced with 0.3 mM IPTG when the OD600 reached 0.8–1.2. After 23 h of incubation at 20 °C, cells were harvested via centrifugation and resuspended in PBS buffer at a ratio of 1 g of cells to 5 mL of buffer. The cell lysates, post-sonication, were loaded onto a Ni-NTA column. This column was washed with a gradient of imidazole concentrations (20 mM Tris-HCl, pH 8.0, with 0, 10, 20, and 40 mM imidazole) to remove non-specifically bound proteins. The proteins were then eluted using an elution buffer (20 mM Tris-HCl, pH 8.0, 500 mM imidazole) when the absorbance at 280 nm was less than 0.1 mg/mL. After dialysis, the purified protein samples were aliquoted and stored at −80 °C.

### 2.3. Substrate LPAT-^iso^K Prediction and Molecular Docking

To predict the three-dimensional (3D) structure of the substrate LPAT-^iso^K, we employed the AlphaFold2 algorithm [23]. Following structural prediction, the LPAT-^iso^K substrate was computationally aligned and docked into the active site of SpSrtA (PDB ID: 7S4O), leveraging the capabilities of AutoDock Vina [24]. The docked conformations and the detailed interaction patterns between LPAT-^iso^K and SpSrtA were thoroughly examined and visualized using PyMOL molecular visualization software (version 2.5.4).

### 2.4. Western Blot Analysis of Protein Ligation Products

Enzymes and substrate protein were expressed and purified from *E. coli* BL21 (DE3) cells. SpSrtA-mediated ligation was conducted using 100 µM biotin-LPETGRRR, 20 µM SpSrtA*, and 50 µM of various purified protein substrates, including sfGFP, MBP, SpyCatcher, and GST. Reactions were performed in a total volume of 100 µL at room temperature overnight in PBS buffer. When SpSrtA* was the sole substrate, no additional protein substrates were added, and reactions proceeded for 1 h. Subsequently, samples were separated on 12% SDS-PAGE gel and transferred onto polyvinylidene fluoride membranes. For detection of biotin modification, membranes were blocked with a solution containing 5% *w*/*v* BSA and 0.1% Tween-20 in PBS for 1 hour at room temperature. The blots were then immersed in streptavidin-HRP (1:5000 dilution) at room temperature for another hour. After the membrane was washed with 0.1% Tween-20 in PBS, it was developed using Clarity™ Western ECL Substrate (1705060, Bio-Rad, Hercules, CA, USA) according to the manufacturer’s protocol. Finally, Coomassie Blue staining and Western blot images were captured using a Bio-Rad ChemiDoc^TM^ Touch Imaging System.

### 2.5. LC-MS/MS and Data Analysis for Identification of Modification Sites 

For this process, 10 µM purified SpSrtA* was incubated overnight at room temperature with 100 µM *Abz*-LPETGK(Dnp)-*NH_2_*, and a control without the enzyme was also prepared. The resulting mixture was precipitated by methanol at −80 °C overnight. After being washed 3 times with pre-cooled methanol, the proteins were resuspended in a solution containing 4 M urea (Sigma-Aldrich, Steinheim, Germany) and 50 mM ammonium bicarbonate (ABC; Sigma-Aldrich, Steinheim, Germany) at a concentration of 5 mg/mL. To reduce disulfide bonds, the proteins were incubated with 10 mM dithiothreitol (Aladdin Biochemical, Shanghai, China) at 37 °C for 45 min. Subsequently, the proteins were treated with 20 mM iodoacetamide (Psaitong Biotechnology, Beijing, China) for 45 min at 25 °C in the dark. The resulting solution was diluted to 1 M urea using 50 mM ABC and then incubated with either mass spectra grade trypsin (Promega, Fitchburg, WI, USA) or Glu-C (Promega, Fitchburg, WI, USA) at an enzyme-to-substrate ratio of 1:50 at 37 °C for 20 h. The resulting peptides were desalted using C18 columns (Waters, Milford, MA, USA) and dried using vacuum centrifugation. 

LC-MS/MS analysis of modified lysine sites was performed on a Q-Exactive Plus Orbitrap mass spectrometer (Thermo Fisher Scientific, Waltham, MA, USA) equipped with a C18 capillary column (75 μm × 15 cm) and an EASY-nLC 1200 system (Thermo Fisher Scientific, Waltham, MA, USA). The peptides were dissolved in solvent A and subjected to gradient elution: 3–10% B for 10 min, 10–37% B for 48 min, 37–100% B for 1 min, 100% B for 5 min, 100–3% B for 1 min, and 3% B for 5 min. Solvent A consisted of 0.1% formic acid (Sigma-Aldrich, Steinheim, Germany) in water (*v/v*), while solvent B comprised 0.1% formic acid and 80% acetonitrile in water (*v/v*). The samples were then subjected to high-collision dissociation (HCD)-based LC-MS/MS. Under positive ion mode, full-scan mass spectra were obtained in the range of 300 to 2000 *m/z* at a resolution of 70,000. The top 20 most intense ions were selected for MS2 analysis using the Orbitrap analyzer with a resolution setting of 17,500.

Raw MS data were processed by MaxQuant software (version 1.5.8.2) integrated with the Andromeda search engine. The sequence of SpSrtA* was imported as a fasta file, and the parameters were set as follows. The additional mass of *Abz*-LPETGK(Dnp)-*NH_2_* was set as a variable modification on protein lysine sites. Methionine oxidation and acetyl N-terminal were set as variable modifications. Carbamidomethyl cysteine was set as a fixed modification. Trypsin/Glu-C was set as the digestion condition with a maximum of 2 missed cleavages. In order to identify modified lysine sites, spectra with an Andromeda score >40, a delta score >8, and a localization score >0.75 were selected and considered as high-confidence sites.

### 2.6. Evaluation of the Efficiency of Lysine-Containing Peptide Tag 

Ten lysine-containing peptide tags were synthesized, each designed with an 11-amino acid motif centered on a lysine residue of SpSrtA*. The motif started from the fifth amino acid preceding the lysine residue. These peptide tags were fused to the N- terminus of sfGFP and assessed with a streptavidin-HRP blotting assay. For this process, 100 µM biotin–LPETGRRR along with 20 µM sfGFP or lysine-containing peptide tag-sfGFP fusion proteins and 20 µM SpSrtA* were incubated in PBS buffer at room temperature for 1 h. This procedure facilitated the formation of stable biotin–LPET conjugates with the protein substrates, where the intensity of biotin labeling served as an indicator of the labeling efficiency of the lysine-containing peptide. Subsequently, the intensity of biotin labeling was visually analyzed for grayscale using the Bio-Rad ChemiDoc™ Touch Imaging System, in accordance with the previously established Western blot protocol.

Through this screening process, the peptide tag containing K111 was identified as the optimal motif, exhibiting the strongest intensity of biotin labeling. The MBP-LPETG fusion construct was synthesized using the pET-28a plasmid as a template, following the Gibson assembly protocol [25]. To evaluate the conversion of conjugates, reactions between 0.1 µM purified sfGFP/K111-sfGFP and 40 µM MBP-LPETG were catalyzed by 20 µM SpSrtA* at room temperature in PBS for 1 h. The reactions resulted in the conjugation of MBP-LPET with sfGFP or K111-sfGFP, forming the ligation products. Western blot analysis was conducted on the substrates and ligation products using anti-GFP primary antibody (1:1000 dilution) and HRP-conjugated goat anti-mouse secondary antibody (1:5000 dilution). Subsequently, Western blot images were captured using the Bio-Rad ChemiDoc^TM^ Touch Imaging System following the previously described Western blot protocol. The quantification of conjugate conversion was performed by calculating the ratio of grayscale values between products and substrates. 

### 2.7. Surface Modification of Living Microorganisms

For *E. coli* (strain DH5α), cells were cultured overnight and then collected in 1.5 mL conical tubes. The cells were washed and resuspended to achieve a final density of approximately 5 × 10^7^ cells per mL in 100 µL PBS. These cells were then incubated with 200 µM biotin–LPETGRRR and 10 µM of either SpSrtA* or its C208A mutant at room temperature for 1 h. Post-incubation, the cells were washed twice with PBS to remove any unbound biotin probes and then incubated with Streptavidin Alexa Fluor™ 488 (1:200 dilution) for 20 min. After being washed and resuspended, cells were analyzed using flow cytometry using the FITC channel to assess biotin modification.

For *Saccharomyces cerevisiae* (*S. cerevisiae*, strain EBY100), cells were cultured overnight in YPD medium. Subsequently, cells were harvested at a final density of about 5 × 10^7^ cells per mL in 100 µL PBS. These cells were then incubated with 400 µM biotin–LPETGRRR and 10 µM SpSrtA* for 1 h. Similar to *E. coli*, the mixture underwent a washing process and was incubated with Streptavidin Alexa Fluor™ 488 (1:200 dilution) for flow cytometry analysis.

### 2.8. Flow Cytometry Analysis of Yeast Surface Display Proteins

The yeast display plasmid pCTCON2 was transformed into EBY100 to generate Aga2p-SpSrtA* yeast. The transformation was performed using a Frozen E-Z Yeast Transformation II Kit (Zymo Research, Irvine, CA, USA) following the manufacturer’s protocol. The transformed yeast was plated on SD solid culture media without uracil and tryptophan for 3 days to obtain transformant colonies. Each individual colony of transformants containing the Aga2p-SpSrtA* plasmid was inoculated into SD media and grown at 30 °C with shaking at 250 rpm for 24 h. To induce protein expression, the yeast cells were cultivated in YPG medium for 20 h after dilution to an OD of 0.2. The expressed cells were pelleted from YPG media at 12,000 rpm for 1.5 min, and the supernatant was discarded. The pellet was washed twice in PBS buffer containing 5% *w*/*v* BSA. Next, the cells were incubated with 400 µM biotin–LPETGRRR for 1 h. To assess Aga2p-SpSrtA* expression, the primary antibody anti-V5 (1:500 dilution) was applied for 1 h. Subsequently, the secondary antibody anti-mouse-Alexa Fluor™ 647 (1:200 dilution) was applied for 20 min. To analyze transpeptidation activity, Streptavidin Alexa Fluor™ 488 (1:200 dilution) was utilized for 20 min, followed by flow cytometry analysis.

### 2.9. Generation of Random Mutant Libraries

Libraries of SpSrtA* mutants were generated using error-prone PCR [26]. The primers used in PCR are listed in Table 1. In brief, for the libraries of SpSrtA*, the template SpSrtA* gene in the pCTCON2 vector was amplified with ep-F and ep-R for 30 cycles using the GeneMorph II random mutagenesis kit (Agilent Technologies, Santa Clara, CA, USA) following the provided protocol. Subsequently, the PCR product was recovered from gel extraction. Then, 200 ng of the recovered product was re-amplified with Homo-F and Homo-R for another 30 cycles under normal PCR conditions. To obtain the linearized pCTCON2 vector, the template pCTCON2-SpSrtA* plasmid was linearized using 1 µL BamHI-HF (R3136V, New England Biolabs, Ipswich, MA, USA), 1 µL NheI-HF (R3131L, New England Biolabs, Ipswich, MA, USA), and 5 µg plasmid DNA in a 50 µL reaction at 37 °C overnight, followed by gel extraction. Subsequently, the error-prone PCR product along with the linearized pCTCON2 vector backbone (5 µg insert: 1 µg vector) were electroporated into electrocompetent EBY100 constitutively expressing Aga2p-SpSrtA* [27]. Electroporation was conducted using a Bio-Rad Gene Pulser XCell, and the transformation efficiency was 10^8^. The electroporated cultures were rescued in 100 mL of SD media supplemented with 100 mg/mL ampicillin and 50 mg/mL kanamycin at 30 °C with shaking at 250 rpm for 2 days. In this study, the activity of SpSrtA* mutants was compared in 100 µM biotin–LPETGRRR for 30 min.

### 2.10. Streptavidin Blot Characterization of SpSrtA* Labeling on Yeast Surface

The Aga2p–SpSrtA* expression on the yeast surface was achieved using the Frozen E-Z Yeast Transformation II Kit, followed by reaction with 1 mM biotin–LPETGRRR in PBS for 1 h, as previously described. The cell pellet was resuspended in PBS buffer containing 2% *v*/*v* SDS and 1% *v*/*v* protease inhibitor cocktail. Next, an equal volume of glass beads was added to the suspension [28]. The mixture was vibrated at 2000 rpm at 4 °C for 1 min, and then cooled on ice for 1 min. The “shake–cool” cycle was repeated 10 times to lyse the yeast cells [28]. The supernatant was collected for Western blot analysis via centrifugation at 12,000 rpm and 4 °C for 2 min. The labeled protein was separated on 12% SDS-PAGE gel. To detect the expression of V5, the blots were incubated with anti-V5 monoclonal antibody (1:5000 dilution) for 1 h, followed by incubation with anti-mouse HRP (1:5000 dilution) as the secondary antibody for an additional 1 h. To detect the biotin labeling signal, the blots were immersed in streptavidin–HRP (1:5000 dilution) at room temperature for another hour, then the chemiluminescence of the blots was captured using the Bio-Rad ChemiDocTM Touch Imaging System.

## 3. Results

### 3.1. Lysine Residue as a Nucleophilic Receptor for SpSrtA*

**Figure 1:**
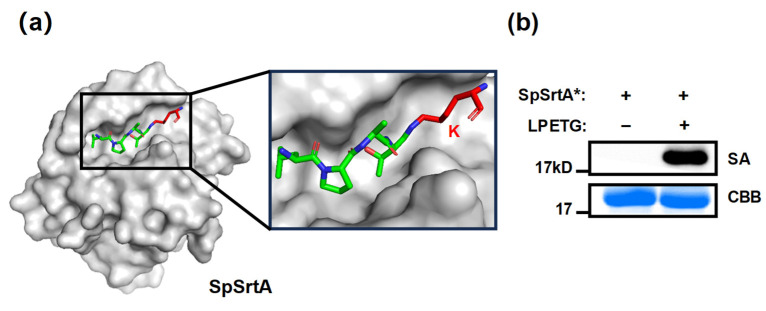
SpSrtA* accepting lysine residues as nucleophilic substrate. (**a**) SpSrtA was simulated with LPAT-^iso^K substrate using Autodock. Protein surface is indicated in gray for SpSrtA (PDB: 7S4O). Simulated peptide substrate LPAT is in green, and ^iso^K is in red. (**b**) SpSrtA* labeled with biotin-LPETGRRR probes. Negative control reaction with biotin–LPETGRRR probes omitted is shown. SA, streptavidin–HRP blotting for biotin-modified SpSrtA* analysis; CBB, Coomassie Brilliant Blue staining of SpSrtA* (~19.7 kDa).

To explore the potential of lysine residues as receptors in transpeptidation, we utilized in silico docking simulations (Autodock) to model the interaction between the substrate LPAT-^iso^K and the natural binding pocket of SpSrtA. The simulation revealed this protein surface conducive to lysine accommodation, characterized by a substantially large and deep pocket (Figure 1a). Previous studies described SrtA’s ability to specifically modify lysine residues in natural pilin motifs, but its activity on lysine is relatively lower compared to N-terminal oligoglycine [17,18]. Significantly, 10 lysine residues in SpSrtA* have been identified as potential sites for labeling. This finding suggests that utilizing SpSrtA* as the sole substrate is a viable method for assessing preliminary enzymatic activity. Due to the absence of an N-terminal glycine, these lysine residues in SpSrtA* act as unique nucleophilic substrates. As expected, SpSrtA* could transfer the LPETG motif to its own lysine residues, forming stable isopeptide bonds (Figure 1b). Herein, we show that SpSrtA* effectively catalyzes ligations involving the ε-amino groups of lysine residues across a range of substrates.

### 3.2. Identification of Lysine Residue Modification Sites in SpSrtA*

**Figure 2:**
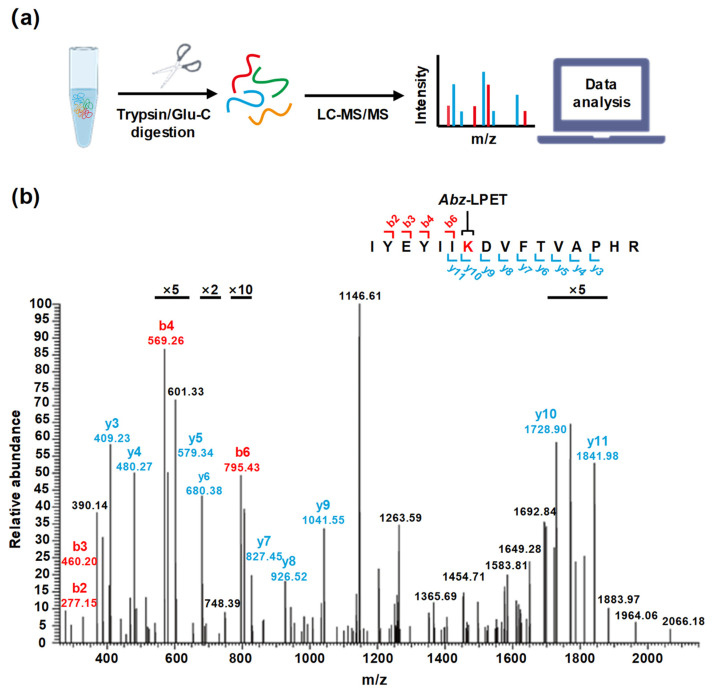
LC-MS/MS results of modified lysine residue sites for SpSrtA*. (**a**) Schematic representation of modification site identification. SpSrtA* was incubated with *Abz*-LPETGK(Dnp)-*NH_2_* peptide, followed by digestion of the peptide segments using trypsin or Glu-C, and subsequent analysis through LC-MS/MS. (**b**) MS2 spectra of K181-containing peptide after trypsin digestion. The y- type ions (blue), b-type ions (red) and other ions (black) elucidated the sequence information of the peptide containing K181. The isolated ion masses confirmed the expected *Abz*-LPET conjugated to the K181 site of SpSrtA* through an isopeptide bond, with K181 labeled in red.

We identified the lysine residue modification sites in SpSrtA* through LC-MS/MS analysis. Purified SpSrtA* was incubated overnight with *Abz*-LPETGK(Dnp)-*NH_2_* peptide, followed by sequential digestion using trypsin or Glu-C (Figure 2a). Ten lysine residues, distributed across the SpSrtA* protein, were identified as potential sites for modification. Given the spatial separation of these lysine residues in the protein sequence, trypsin and Glu-C digestion were essential to ensure a comprehensive analysis of all lysine-inclusive peptide segments [29,30]. Our LC-MS/MS analysis detected an additional mass increase of 559.26 Da, matching the mass of *Abz*-LPET, unique to peptides containing lysine. In the MS2 spectrum, fragmentation of the modified protein backbone generated several daughter ions, conclusively indicating that the modifications were confined to the ε-amino groups of lysine residues (Figure 2b). Digestion fragments validated modification at four distinct lysine sites—K181, K201, K220, and K224 (Table 2). The modification mechanism may involve two catalytic steps: (i) There is specific recognition and cleavage of the amide bond between threonine and glycine, resulting in an acyl-enzyme intermediate. (ii) The intermediate thioester is subjected to nucleophilic attack by the exposed lysine side chain within SpSrtA*, culminating in the generation of a ligation product through the formation of a stable isopeptide bond.

### 3.3. Isopeptide Ligation with Diverse Proteins Lacking N-Terminal Glycine

**Figure 3:**
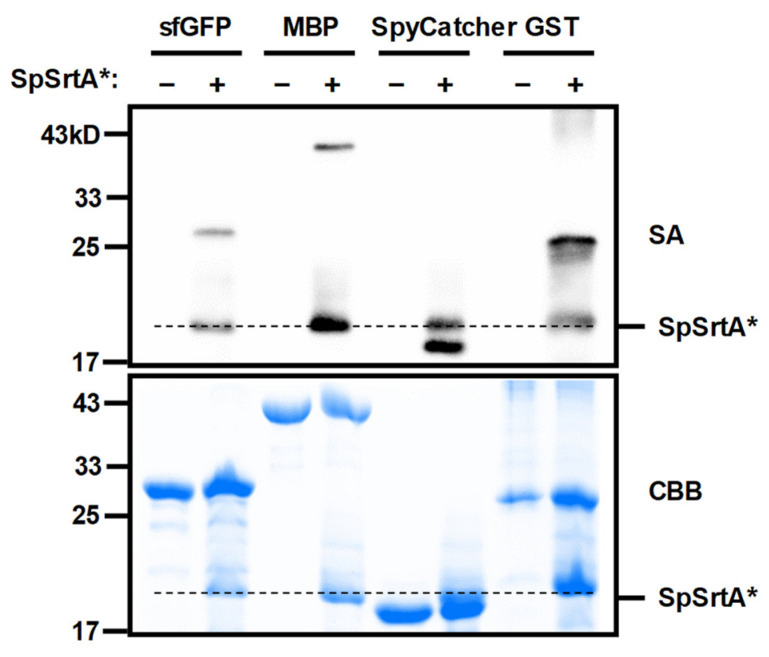
Modification of various protein substrates by SpSrtA*. Western blot analysis was performed to evaluate modification of various substrate proteins with biotin–LPETGRRR catalyzed by SpSrtA*. No sortase enzyme was used as negative control. SA, streptavidin-HRP blotting for biotin-modified protein analysis; CBB, Coomassie Brilliant Blue staining of various protein substrates and SpSrtA*. Bands corresponding to protein substrates sfGFP (~26.8 kDa), MBP (~42.0 kDa), SpyCatcher (~17.9 kDa), and GST (~26.8 kDa) are shown. Dotted lines indicate the presence of SpSrtA* (~19.7 kDa).

We utilized SpSrtA* for isopeptide ligation across an array of protein substrates that are either intrinsic to or commonly used in bacterial systems. These proteins, characterized by the absence of N-terminal glycine and the presence of lysine residues, are not suitable for traditional SML methods [31]. In our study, we initially used biotin–LPE–GRRR as a model donor peptide. This peptide, along with the protein substrates, was incubated with SpSrtA*. The subsequent biotin labeling signals on these proteins were captured by using streptavidin–HRP in Western blot analysis. For comparison purposes, the reactants were visualized using Coomassie Blue staining (Figure 3). We first confirmed that LPETG peptide could be conjugated to the ε-amino groups of lysine residues in various protein substrates. Since most proteins naturally possess a relative abundance of exposed lysine residues, these modifications extend beyond a single moiety attachment at a protein’s terminus. Overall, the use of SpSrtA* for protein modification proved to be a robust method, particularly for labeling lysine residues in a wide array of proteins of interest. 

### 3.4. Screening for Optimal Lysine-Containing Peptide Tag

**Figure 4:**
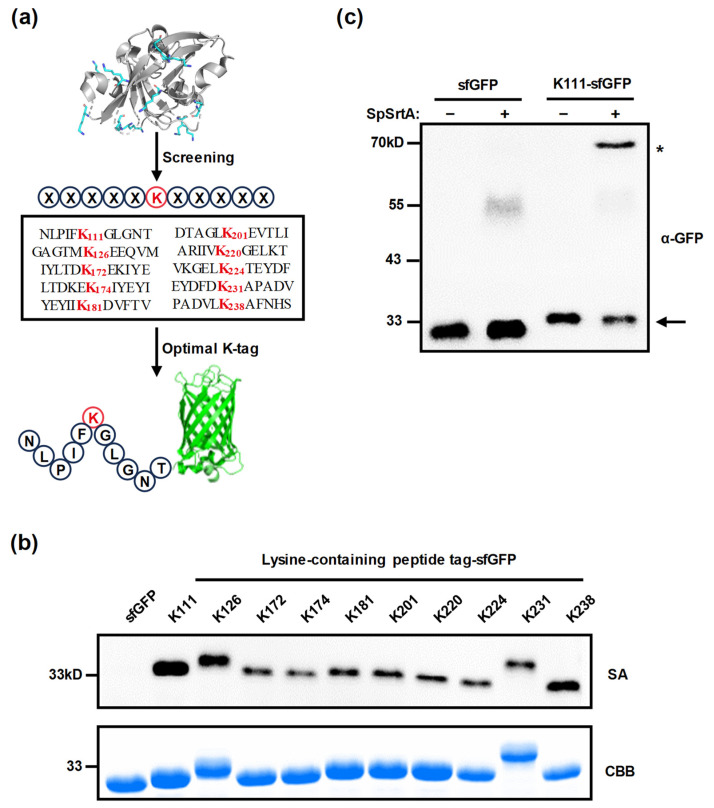
Enhancement of protein modification efficiency by incorporating lysine-containing peptide tag. (**a**) Schematic representation of screening process for optimal lysine-containing peptide tag derived from SpSrtA*. The lysine residue at the center of the peptide tag is labeled in red. (**b**) Analysis of labeling efficiency of sfGFP, without or with lysine-containing peptide tag conjugation, catalyzed by SpSrtA*. K111 tag was found to be the best lysine-containing peptide tag for SpSrtA* modification. SA, streptavidin-HRP blotting for biotin-modified protein analysis; CBB, Coomassie Brilliant Blue staining. Bands corresponding to sfGFP (~26.8 kDa), K111-sfGFP (~28.0 kDa), K126-sfGFP (~28.0 kDa), K172-sfGFP (~28.0 kDa), K174-sfGFP (~28.2 kDa), K181-sfGFP (~27.9 kDa), K201-sfGFP (~27.9 kDa), K220-sfGFP (~28.0 kDa), K224-sfGFP (~28.1 kDa), K231-sfGFP (~28.1 kDa), and K238-sfGFP (~28.0 kDa) are shown. (**c**) Analysis of conversion of sfGFP without or with optimal lysine-containing peptide tag using SpSrtA*. Conversion was quantified by comparing grayscale values of products and substrates. K111-sfGFP shows conversion of 57%. Arrow indicates sfGFP or K111-sfGFP. Asterisk (*) indicates conjugation product. α-GFP, anti-GFP blotting of sfGFP substrates and product conjugates.

To enhance the protein modification efficiency for target proteins, we focused on screening out the optimal lysine-containing peptide tag derived from SpSrtA* (Figure 4a). Since SpSrtA* acted as a preferred substrate for SML, we then sought a good lysine-containing motif in SpSrtA*. We added every 11-amino acid motif, five residues on each side around the lysine residues in SpSrtA*, at the N-terminus of target protein sfGFP. Previous observations indicated that sfGFP is a suboptimal substrate for SpSrtA* (Figure 3). The modified sfGFP constructs were expressed and purified. Then, the sfGFP constructs were incubated with biotin–LPETGRRR probes in the presence of SpSrtA*, and the labeling efficiency was assessed by using streptavidin-stained Western blot. Remarkably, the modification efficiency was significantly enhanced by incorporating just one tag onto the target protein sfGFP, as compared to sfGFP without any tag fusion (Figure 4b). Notably, the peptide tag containing lysine residue K111 (NLPIFKGLGNT) emerged as the most effective nucleophilic tag. For a more precise evaluation of conjugate conversion, we replaced the biotin–LPETGRRR probe with MBP-LPETG fusion protein as the donor substrate. Conjugation of MBP-LPET with K111-sfGFP was observed via anti-GFP blotting. The sfGFP construct with the K111 tag resulted in a significant conversion of 57%, while no conversion was observed in wild-type sfGFP (Figure 4c). Altogether, integrating the lysine-containing peptide tag proved to be a highly effective strategy for modifying target protein. 

### 3.5. Application of SML for Labeling of Living Microorganism Surfaces

**Figure 5:**
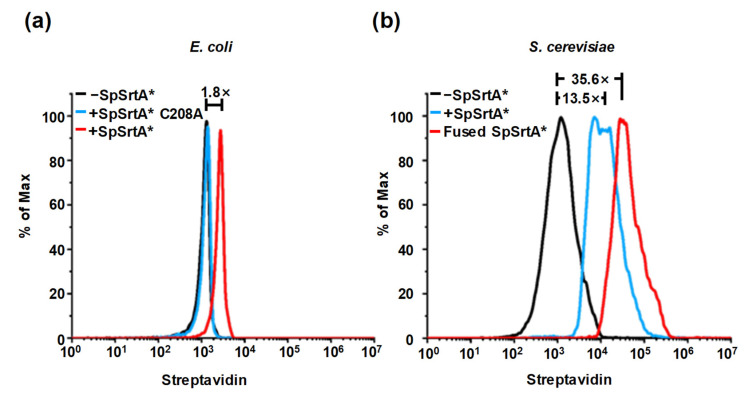
Surface labeling of living microorganisms’ surface with SpSrtA*. (**a**) Flow cytometry analysis of *E. coli* surface labeling with SpSrtA* (red), inactive SpSrtA* C208A (blue), and negative control reaction with PBS buffer (black). SpSrtA* shows 1.8-fold improvement compared to SpSrtA* C208A and negative control. (**b**) Flow cytometry analysis of *S. cerevisiae* surface labeling with SpSrtA* (blue) and negative control reaction with PBS buffer (black). SpSrtA* expressed on *S. cerevisiae* surface by fusing with its surface protein Aga2p (red) instead of adding exogenous SpSrtA*, is also shown for comparison. SpSrtA* increased 13.5-fold compared to negative control and fused SpSrtA* further improved 2.64-fold.

We explored the innovative use of SpSrtA* for modifying lysine residues on protein substrates, particularly focusing on the surfaces of living microorganisms. We first used SpSrtA* to label microorganisms traditionally considered incompatible with SML, such as those lacking proteins with N-terminal glycine, alanine, or serine [32,33]. SpSrtA* was added to the microorganism suspension with biotin LPETGRRR for 1 h incubation; after the enzyme reaction, Streptavidin Alexa Fluor™ 488 was added to stain the cells, followed by flow cytometry analysis. C208 in SpSrtA* is regarded as a conserved catalytic site [22]. When we used either an inactive variant of SpSrtA* (SpSrtA* C208A) [22] or PBS buffer as a negative control, the biotin labeling was nearly the same. However, with active SpSrtA*, we successfully achieved a 1.8-fold increase in labeling on the surface of *E. coli* (Figure 5a). More strikingly, the surface of *S. cerevisiae* presented up to a 13.5-fold increase in labeling efficiency (Figure 5b). This may be because the *S. cerevisiae* surface proteins, which are present in much higher abundance than those of *E. coli*, could be substrates for SpSrtA*. To further improve the labeling efficiency, we engineered a fusion of SpSrtA* with the C-terminus of Aga2p protein, a surface protein of yeast linked covalently to the cell wall-anchoring Aga1p protein. The intention with this fusion was to position SpSrtA* in close proximity to the surface proteins, thereby prioritizing and enhancing the labeling efficiency. We successfully labeled the *S. cerevisiae* surface with another 2.64-fold increase (Figure 5b).

### 3.6. Directed Evolution of SpSrtA* for Enhanced Surface Protein Labeling

**Figure 6:**
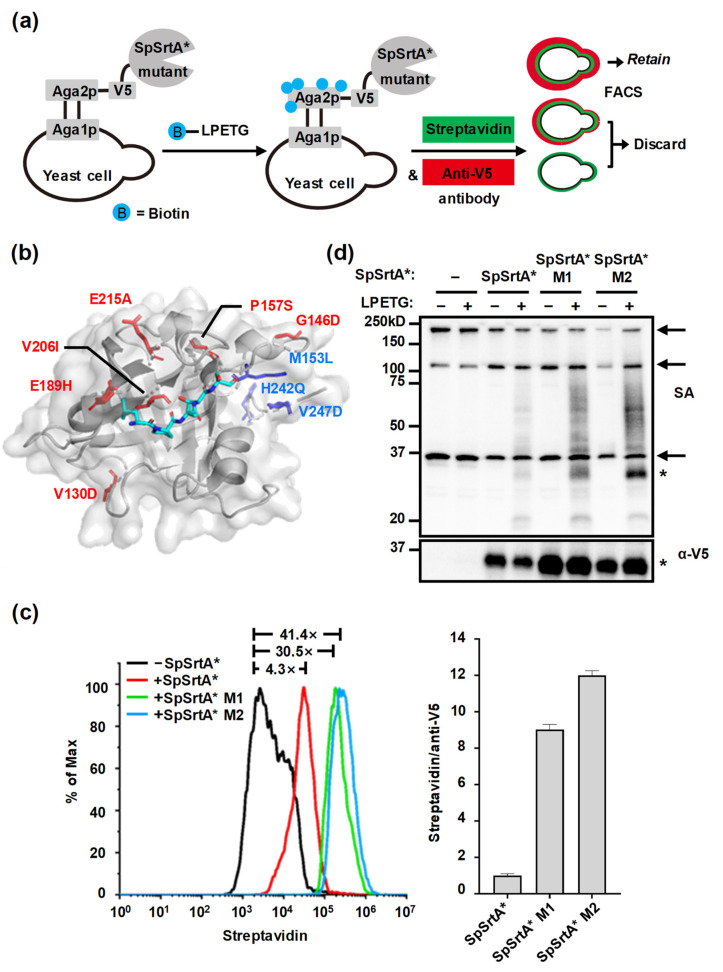
Directed evolution of SpSrtA* for modifying proximity protein on yeast surface. (**a**) Schematic representation of labeling and selection used to evolve SpSrtA*. Two-dimensional fluorescence-activated cell sorting was used to enrich cells displaying highest activity/expression (streptavidin/anti-V5) ratio. (**b**) Labeling of mutant SpSrtA sites in complex with its substrate peptide (PDB: 7S4O). Cyan: peptide substrate LPATS; blue: residues from previously reported SrtA triple-mutant [2]; red: residues evolved in SpSrtA* M2 (V130D, G146D, M153L, P157S, H242Q, and V247D). (**c**) Left, histograms of SpSrtA* mutant transpeptidation activity on yeast surface compared by flow cytometry. Transpeptidation activity is defined as mean fluorescence intensity of streptavidin. Right, mean of activity/expression ratio normalized to SpSrtA* mutants on yeast surface compared by flow cytometry. Data in bar graph indicate mean ± SD of three independent experiments. (**d**) SpSrtA* mutant labeling on yeast surface with biotin–LPETGRRR by Western blot. SA: streptavidin-HRP blotting showing biotin labeling on yeast surfaces; α-V5: anti-V5 blotting showing expression of Aga2p-SpSrtA* and mutants (~33.4 kDa). Arrow indicates endogenously biotinylated proteins; asterisk (*) indicates Aga2p-SpSrtA* and mutant proteins (~33.4 kDa).

We developed a high-throughput yeast display screening method for the directed evolution of SpSrtA* (Figure 6a). Using error-prone PCR, we mutated the SpSrtA* template, creating a library of 10^8^ transformants on the yeast surface, and incorporated a V5 tag between SpSrtA* and Aga2p protein (Figure 6a). The V5 tag, positioned on the cell surface, facilitated the assessment of Aga2p-SpSrtA* expression levels. This was achieved by using an anti-V5 antibody, followed by staining with Alexa Fluor™ 647 secondary antibody. The reaction was initiated by adding biotin–LPETGRRR probes for 1 h incubation. After incubation, Streptavidin Alexa Fluor™ 488 was used to bind the biotinylated substrates, providing an indicator of transpeptidation activity based on the fluorescence intensity of Alexa Fluor™ 488. After fluorophore staining, cells with dual fluorescence levels displaying the highest activity/expression (streptavidin/anti-V5) ratio were sorted via flow cytometry and amplified through culturing. Following six rounds of sorting and enrichment, there was a significant increase in SpSrtA* triple-mutant (SpSrtA* M1: V130D/G146D/P157S) (Figure 6b,c). Building upon this, we generated a second mutant library from SpSrtA* M1, which was enriched in a highly active hexa-mutant (SpSrtA* M2: V130D/G146D/M153L/P157S/H242Q/V247D) (Figure 6b,c). To better compare the labeling efficiency of these mutants, we reduced the reaction substrate concentration and reaction time. This adjustment resulted in a modest 4.3-fold increase in labeling efficiency of SpSrtA* compared to the negative control, and the transpeptidation activity of SpSrtA* M1 and M2 improved by 7.1-fold and 10-fold, respectively, compared to SpSrtA* (Figure 6c). Additionally, we observed up to a 12-fold improvement in the activity/expression ratio for SpSrtA* M2 (Figure 6c). Beyond activity enhancement, SpSrtA* M2 increased the preference for the fusion protein Aga2p and used Aga2p as the selective target, as shown by streptavidin blotting analysis (Figure 6d). In conclusion, through directed evolution using yeast display, the SpSrtA* mutants achieved higher labeling efficiency and higher specificity for fused substrates.

## 4. Discussion

In conclusion, we developed a novel strategy for modifying lysine residues using SpSrtA*, significantly enriching the selection of protein bioconjugation techniques. Our method not only extends the scope of substrates beyond the conventional N-terminal oligoglycine but also establishes SpSrtA* as a robust catalyst for isopeptide ligation in a variety of protein substrates. We explain that a possible mechanism for stable isopeptide bond formation involves acyl-enzyme intermediate being attacked by ε-amino groups of lysine residues. Furthermore, we successfully identified modified lysine sites in SpSrtA* using LC-MS/MS and isolated an optimal lysine-containing peptide tag (NLPIFKGLGNT) derived from SpSrtA*. In particular, incorporating this tag into the target protein sfGFP resulted in over 50% modification efficacy. Building on this foundation, we applied SpSrtA* for the labeling of microorganism surface proteins, achieving up to a 13.5-fold increase in efficiency. In pursuit of further enhancement, we fused SpSrtA* onto a surface protein, thereby realizing a 2.64-fold improvement in labeling efficiency. Upon recognizing the suboptimal substrate affinity of the fused surface protein for SpSrtA*, we carried out a directed evolution strategy using yeast display. After conducting two rounds of directed evolution, we successfully isolated the SpSrtA* M2 variant, which exhibited up to a 10-fold enhancement in labeling efficiency toward the surface protein.

This method effectively overcomes two major limitations inherent in the canonical native ligation catalyzed by SrtA: (1) complex engineering modifications are required, and protein modification can only occur at the N-terminus or C-terminus of the protein, and (2) normally only one donor is attached to the target protein [18]. Our methodology demonstrates significant advancements, but there are still limitations, particularly with regard to the factors influencing the modification of lysine residues with SpSrtA*, which could be improved. First, the position of the lysine-containing peptide tag domain within a protein may affect lysine exposure and solvent accessibility, impacting kinetics and yield. Second, modification efficiency is likely to be influenced by the precise sequence of these peptide tags. We expect that screening this sequence through a library would help to further optimize it for enhanced performance. Moreover, fused multiplicative tags could enable complete substrate transformation. Finally, enzyme activity plays a crucial role in governing the reaction. The commonly utilized SrtA for SML is derived from *Staphylococcus aureus* (SaSrtA) and has been engineered to increase its activity by hundreds of folds [9]. SpSrtA* can also promote activity toward any protein of interest through evolution, combined with high-throughput screening methods such as phage display [34], bacterial display [35], yeast display [36], and SAMDI technology [37], which facilitate the screening of exceptional mutants from a vast library. 

The commonly used SrtA serves as a versatile tool for chemoselective ligation and modification, conjugating proteins and peptides [38], as well as diverse biomolecules [39,40,41,42,43], through SML. In our study, SpSrtA* targeted lysine residues, inspiring the exploration of additional applications through SML. Here, we provide specific enzymes to use for antibody–drug conjugate (ADC) generation such as the transglutaminase [44], glycotransferase [45], and formylglycine-generating enzymes [46]. Reactive lysine residues naturally exist in the Fc region of IgG antibodies, which makes it easier for SpSrtA* to catalyze toxin conjugations and form potent ADCs. Recently, a SrtA-based method known as enzyme-mediated proximity cell labeling (EXCELL) has been successfully employed to monitor cell–cell interactions (CCIs) by labeling naturally occurring N-terminal glycine on the cell surface [47]. However, N-terminal glycine constitutes only about 5% of surface proteins, and SpSrtA*, which targets the relative abundance of lysine residues, is clearly a more appropriate CCI labeling tool. Pupylation-based interaction tagging (PUP-IT) primarily targets lysine side chains rather than the N-terminal amino group targeted by EXCELL [48,49]. Similar to PUP-IT, SpSrtA* is able to capture transient and weak protein–protein interactions. Significantly, gene editing of gut microbiota is still challenging [50]. We plan to label the gut microbiota with SpSrtA* fused with an N-terminal LPETG. After labeling, the gut microbiota will be transplanted into the intestine, and we will study the interactions between transplanted and endogenous microbiota or intestinal epithelial cells. Further research endeavors could focus on developing universal lysine modification tools to detect complex interactions and cell communication among microorganisms in order to uncover the mysteries of biological processes.

## Figures and Tables

**Table 1:** Primer sequences used in random mutant libraries of SpSrtA*.

Primer	Sequence from 5′ to 3′
ep-F	TGGAGGAGGCTCTGGTGCTAGC
ep-R	TAGTCTGGAACGTCGTATGGGTAGGATCC
Homo-F	CAAGGTCTGCAGGCTAGTGGTGGAG-GAGGCTCTGGTGCTAGC
Homo-R	TGTTGTTATCAGATCTCGAGCTATTAGGCATAGTCTGGAACGTCGTATGGGTAGGATCC

F, forward; R, reverse.

**Table 2:** LC-MS/ MS analysis of peptides.

Position	Sequence	Calculated Mass (Da)	Precursor Mass (Da)
181	IYEYII**K**DVFTVAPHR	2522.32	2522.32
220	IIV**K**GELK	1457.85	1457.85
224	GEL**K**TEYDFDKAPADVLK	2597.29	2597.29
181	YII**K**DVFTVAPHRVDVIDDTAGLKE	3372.76	3372.76
201	YIIKDVFTVAPHRVDVIDDTAGL**K**E	3372.76	3372.76

## Data Availability

Data are contained within the article.
